# Iranian nurses’ attitudes towards the necessity and barriers to developing nurse prescribing roles

**DOI:** 10.1186/s12912-021-00700-5

**Published:** 2021-09-23

**Authors:** Azam Naderi, Maryam Janatolmakan, Rostam Jalali, Bahare Andayeshgar, Alireza Khatony

**Affiliations:** 1 School of Nursing and Midwifery, Kermanshah University of Medical Sciences, Kermanshah, Iran; 2 Clinical Research Development Center, Imam Reza Hospital, Kermanshah University of Medical Sciences, Kermanshah, Iran; 3 School of Nursing and Midwifery, Kermanshah University of Medical Sciences, Kermanshah, Iran; 4 School of Health, kermanshah University of Medical Sciences, kermanshah, Iran; 5 Infectious Diseases Research Center, Kermanshah University of Medical Sciences, Kermanshah, Iran; 6 Social Development and Health Promotion Research Center, Health Institute, Kermanshah University of Medical Sciences, Kermanshah, Iran

**Keywords:** Intensive care, Nursing prescribing, Attitude, Barriers

## Abstract

**Background:**

Prescribing medication by nurses as an approach to rational drug prescription has been proposed in many countries. Nursing prescribing is an effective measure in the management of critically ill patients admitted to intensive care units (ICU). This study investigated the attitude of ICU nurses towards the necessity and the barriers to developing nursing prescribing.

**Materials and methods:**

In this cross-sectional study, 136 ICU nurses were included by stratified random sampling. The data collection tool was the researcher-made questionnaire. Cronbach’s alpha method was used to evaluate the reliability of the instrument. The validity of the instrument was also verified by the content validity method. To collect the data, the researcher referred to the nurses’ workplace and provided them with a questionnaire and collected it after completion.The collected data were analyzed by IBM SPSS 16 using descriptive and inferential statistics.

**Results:**

It was revealed that 58.8 % of nurses were familiar with the term ‘nursing prescribing’; a majority (92.1 %) of whom considered it vital to develop this role in the ICU. Moreover, 86 % (n = 98) of the nurses assumed that it is possible to implement this role in ICU. The most potential barriers to its implementation were lack of legitimacy, disapproval of physicians, and the reluctance of nursing managers.

**Conclusions:**

Most nurses maintained a positive attitude towards nursing prescribing; hence, its legitimacy seems vital in ICUs. For the development of the ‘nurse prescribing’ role, the Nursing System Organization may be helpful.

## Introduction

Non-physician prescribed medications and non-medical prescribing (NMP) are among the solutions in developed countries to meet the increased need for health care [1]. Thus, it would be a successful innovation to improve the efficacy of the services and achieve faster access to medication and patient-centered care [2, 3]. Nurses are the most prominent group among non-medical prescribers [4]. During its growth and development, nurse prescribing has a long and gradual progression of implementation in different countries at different times.NMP was proposed in 1969 in Idaho, United States, and then developed over time [5, 6]. Until 2019, thirteen European countries, including England, Ireland, Netherlands, and Sweden, had ratified some nurse prescribing laws [7]. The basic policy in nurse prescribing in these countries includes improved patient care, utilizing professional nursing skills, and supporting teamwork [8].

Previously, prescribing medication was among the duties of the physicians [9, 10], and the opposition of the physician was the most critical barrier to the implementation of the “nursing prescribing” [11]. Various reports indicated that the initial objection of the physicians has decreased, as it contributes to the improved access of the patients to medication, patient safety, and patient-centered care [12].

A qualitative study with physician participants revealed that nurse prescribing was beneficial for patients and health professionals, reduced physicians’ workload, and improved patient comfort; hence, they approved it [13]. Jiao et al. (2018) performed cross-sectional studies in theUS during 2006 and 2012. They compared the viewpoints of physicians and nurses to investigate the quality of medication prescribed by physicians and nurses. Their study indicated no significant difference in the quality of care between physicians and nurses. In other words, it was shown that the prescription quality was similar between nurses and residents [14].

From the legal perspective, Iranian nurses are not certified to prescribe medications. However, a study by Darvishpour et al. (2014) revealed that despite the legal prohibition, nurses prescribe medication in most wards (particularly emergency wards and intensive care units) [15]. In emergencies, providing urgent services is vital to save the life of the patient [16]. The majority of studies in this regard suggest that most nurses are interested in developing such a role; since it would be formalizing and publicizing an act, which is being done informally for years [17–19]. Darvishpour et al. (2016) showed the positive attitude of nurses towards the role of nursing prescribing, which plays a vital role in its institutionalization [15]. Nevertheless, the inadequate knowledge and negative attitude of medical professionals are considered direct obstacles for implementing the nurse prescribing plan [20], while the positive attitude of nurses plays a crucial role in the implementation of the nurse prescribing [6]. Since the viewpoints of Iranian ICU nurses on the ‘nursing prescribing’ role has not been examined so far, the present study aims to investigate the attitudes of Iranian nurses on the subject and the potential barriers to implementing this role.

This study intended to find the answers to the following questions: How familiar are nurses with the ‘nursing prescribing’ term? Why is it necessary to develop the role of nurse prescribing for ICU nurses? Is it possible to implement the ‘nursing prescribing’ function in Iran? Does a relationship exist between the attitude of nurses on the necessity of developing the ‘nurse prescribing’ role with their clinical experience and level of education? Is medication prescription by the nurses related to their work experience and level of education?

## Materials and Methods

### ***Study design***

The present research was a descriptive-analytic cross-sectional study conducted from April 2019 to March 2020 at the Kermanshah University of Medical Sciences (Kermanshah, Iran).

### Sample and sampling method

The study population in this research included all nurses who worked in ICUs of hospitals affiliated to Kermanshah University of Medical Sciences. Using Cochran’s formula with 95 % confidence, the ideal sample size (n = 136) was calculated. Sampling was based on the stratified random sampling method. The inclusion criteria for the study consisted of a bachelor’s degree or higher in nursing, a minimum of one year of working experience in ICU, and the willingness of the nurses to participate in the study. The incomplete questionnaires were not analyzed.

### Data collection tool

A four-section questionnaire was used as the data collection tool. The researcher designed the first two sections. The first section included demographic variables, including age, gender, work experience, clinical experience in ICU, marital status, and education level.

The second section included five general questions on nurse prescribing in the ICU. This section was scored as yes (score 1) or no (score 0). The third and fourth sections of the questionnaire were inspired by Lock Wood et al. (2008) [21].

The third section included questions on the necessity of nurse prescribing in the ICU, consisting of 15 questions with a total score ranging between 15 and 60. The fourth section was related to the potential barriers of implementing nurse prescribing in the ICU, consisting of 9 questions with scores ranging from 9 to 36. The third and fourth sections were scored based on the four-point Likert score, including strongly disagree (1), disagree (2), agree (3), and strongly agree (4). Participants chose the level of their agreement with the statements using these options. The researcher examined the reliability and validity of the Persian version of this questionnaire.

Regarding the reliability of the study, a total of 30 nurses working in the ICU reviewed the questionnaire. Moreover, the internal consistency of the questionnaire was evaluated using Cronbach’s alpha, and for the second, third, and fourth sections of the questionnaire, it was calculated as 77 %, 78 %, and 79 %, respectively. The content validity index was used to check the validity of the measurement. Thus, the questionnaire was submitted to 12 nursing faculty members and experienced nurses familiar with the research method. They reviewed the questions to check for relevance, comprehensibility, and simplicity. In editing the questionnaire, their corrective comments were considered. This questionnaire examined the clinical attitude of nurses towards nurse prescribing and its barriers. Its reliability was assessed and confirmed using the test-retest method (r = 0.72). The questionnaire’s validity was evaluated and approved by a panel of three nursing experts.

### Data collection method

Following the approval of the study by the Ethics Committee of the university, the researcher referred to the nursing stations of affiliated hospitals of the university to receive and mark the list of ICU nurses. Next, using a random number table, the sample size proportional to the number of nurses of each hospital was determined. Then, the researcher referred to these nurses and explained the study objectives. If they were willing to participate in the study, the researcher provided them with a questionnaire. In the event of the disinclination of a nurse to participate in the study, the nurse was replaced by the preceding or following nurse on the list.

### Statistical analysis

Following the completion of the questionnaire, the data were imported to IBM SPSS 16. The data were analyzed using descriptive analysis (i.e., frequency distribution tables, mean, variance, standard deviation) and inferential analysis (chi-square test). The *p*-values less than 0.05 were considered statistically significant.

### Ethical considerations

 The research ethics committee of Kermanshah University of Medical Sciences approved the study ( IR.KUMS.REC.1397.890). Written informed consent was obtained from all of the participants. Also, the participants were informed of the confidentiality of their information.

## Results

Overall, 114 nurses completed the questionnaire (response rate = 83.21 %). The average age of the participants was $35.5\pm 7.6$ years. Moreover, 60.5 % (n = 69) of the participants were female. The average overall working experience and ICU work experience were $10.8\pm 6.3$ and 8.5 ± 6.8 years, respectively. Moreover, 71.9 % (n = 82) of the participants had a bachelor’s degree. Considering job position, 76.3 % (n = 87) of the participates were nurses.

 Answers to the first question regarding familiarity with the subject of ’nurse prescribing’ indicated that 58.8 % (n = 67) of the participants were familiar with this term, while 41.2 % (n = 47) of the participants heard it for the first time. Moreover, 67.5 % (n = 77) of the participants had the experience of prescribing medication for their patients without the physician’s permission. Also, 92.1 % (n = 105) of the participants considered it vital to develop this role in the ICU.

Answers to the second question (i.e., determining the viewpoints of nurses on the necessity of developing nurse prescribing roles in the ICU) revealed that 92.1 % (n = 105) of the participants considered the development of this role necessary. Almost all of the answers to items regarding the positive effects and necessity of this role were positive. In other words, more than 90 % of the nurses believed that the ‘nurse prescribing’ role leads to the promotion of the nursing profession, provides comprehensive care, easier access to health care services, enhanced quality of nursing care, and improved use of nursing skills. Moreover, 85.9 % (n = 98) of the participants considered this role driven from the medical field, while 64.1 % (n = 73) of them believed to have the required educational competence for prescribing medication (Table 1).

**Table 1:** Attitudes of ICU nurses towards the need to develop the role of nurse prescribing

Nurses’ views on nurse prescribing	Totally disagree & disagree n (%)	Agree & totally agree n (%)
1. Nurse prescribing is derived from the roles of physicians	16 (14.1)	98 (85.9)
2. Nurse prescribing is an essential nursing care	8 (7.1)	106 (92.9)
3. Nurse prescribing should only be for advanced nurses	60 (52.7)	54 (47.3)
4. Nurse prescribing leads to nursing professional development	4 (3.5)	110 (96.5)
5. Nurse prescribing improves the provision of comprehensive nursing care	10 (8.8)	104 (91.2)
6. This role needs to be expanded due to the benefits of nurse prescribing	7 (6.2)	107 (93.8)
7. Nurse prescribing should be for all levels of nursing	20 (17.6)	94 (82.4)
8. I feel I am not educationally competent to prescribe	73 (64.1)	41 (35.9)
9. The role of prescribing medications should not be given to nurses	95 (83.3)	19 (16.7)
10. Nurse prescribing improves the quality of care for patients	72 (63.2)	42 (36.8)
11. Nurse prescribing lead to easier access to patient care services	10 (8.8)	104 (91.2)
12. Continued care is provided following a Nurse prescribing	8 (7.0)	106 (93.0)
13. It is possible to make better use of nurses’ skills following a Nurse prescribing	4 (3.5)	110 (96.5)
14. Nurse prescribing increases the professional independence of nurses	7 (6.2)	107 (93.8)

Also, answers to the third question regarding ‘possibility of the implementation of nurse prescribing in Iran’ indicated that 86 % (n = 98) of the participants believed it to be possible in the ICUs, and 95.6 % (n = 109) of them agreed on nurse prescribing after fulfilling the requirements (Table 2).

**Table 2:** General questions about nurse prescribing

Questions	Yes n (%)	No n (%)
1. Are you familiar with the term nurse prescribing?	67 (58.8)	47 (41.2)
2. Have you ever prescribed medication to your patients in the ICU without a doctor’s permission?	77 (67.5)	37 (32.5)
3. Do you think there is a need to develop the role of nurse prescribing in the ICU	105 (92.1)	9 (7.9)
4. Is it possible to develop the role of nursing in the ICU?	98 (86.0)	16 (14.0)
5. Do you think that ICU nurses are able to safely and correctly prescribe Medication for patients after fulfilling the requirements?	109 (95.6)	5 (4.4)

The responses to the fourth question of this study on ‘surveying the viewpoints of nurses on the potential barriers to nurse prescribing in the ICU’ indicated that the most common barriers include the legal prohibition of this category in Iran (96.5 %), lack of the physician’ (95.6 %), lack of nursing managers’ support (95.5 %), lack of knowledge (94.8 %), and fear of the potential legal consequences (93.8 %), respectively (Table 3).

**Table 3:** Attitudes of ICU nurses towards potential barriers to nurse prescribing

Barriers to Nurse prescribing	Totally disagree & disagree n (%)	Agree & totally agree n (%)
1. Lack of adequate financial support	22(19.7)	92(80.3)
2. Lack of training	6(5.2)	108(94.8)
3. Lack of nurses’ knowledge about nurse prescribing	18(15.8)	96 (84.2)
4. Lack of clinical experience	12(10.5)	102 (89.5)
5. Illegality of this practice in Iran	4(3.5)	110(96.5)
6. Lack of support from nursing managers	5(4.4)	109(95.6)
7. Doctors’ opposition	4(3.5)	110(96.5)
8. No allocation of rewards and benefits	16(14.0)	98 (86.0)
9. Fear of lawful consequences	7(6.2)	107(93.8)

The obtained results indicated no significant relationship between the work experience of nurses and their attitude towards the necessity of developing the ‘nurse prescribing’ role, and the answer to none of the questions showed a significant relationship with the nurses’ working experience (Table 4). On the other hand, a statistically significant relationship was revealed between the educational level of the nurses and their viewpoint regarding the necessity of developing the “nursing prescribing” role. In other words, nurses with master’s degrees were more familiar with the term ‘nursing prescribing’ compared with nurses with bachelor’s degrees (*p* = 0.028, X^2^ = 4.83) (Table 5).

**Table 4:** Relationship between nurses’ attitudes on the second section of the questionnaire with their clinical work experience

General questions about nurse prescribing	Responses	Clinical experience under 15 years n (%)	Over a 15 year of clinical experience n (%)	
1. Are you familiar with the term nurse prescribing?	No	35(39.8)	12(46.2)	*X*^*2*^ = 0.337 *P* = 0.561
	Yes	53(60.2)	14(53.8)	
2. Have you ever prescribed medication to your patients in the ICU without a doctor’s permission?	No	31(35.2)	6(23.1)	*X*^*2*^ = 1.35 *P* = 0.245
	Yes	57(64.8)	20(76.9)	
3. Do you think there is a need to develop the role of nurse prescribing in the ICU?	No	9(10.2)	0(0.0)	*X*^*2*^ = 2.88 *P*^*f*^=0.116
	Yes	79(89.8)	26(100.0)	
4. Is it possible to develop the role of nursing in the ICU?	No	14(15.9)	2(7.7)	*X*^*2*^ = 1.123 *P*^*f*^=0.357
	Yes	74(84.1)	24(92.3)	
5. Do you think that ICU nurses are able to safely and correctly prescribe Medication for patients after fulfilling the requirements?	No	5(5.7)	0(0.0)	*X*^*2*^ = 1.54 *P*^*f*^=0.584
	Yes	83(94.3)	26(100)

**Table 5:** Relationship between nurses’ attitudes on the second section of the questionnaire with their level of education

General questions about nurse prescribing	Responses	Bachelor degree n (%)	Master degree n (%)	
1. Are you familiar with the term nurse prescribing?	No	39(47.6)	24(75)	*X*^*2*^ = 4.83 *P* = 0.028
	Yes	49(59.8)	8(25)	
2. Have you ever prescribed medication to your patients in the ICU without a doctor’s permission?	No	33(40.2)	28(87.5)	*X*^*2*^ = 8.08 *P* = 0.004
	Yes	74(90.2)	4(12.5)	
3. Do you think there is a need to develop the role of nurse prescribing in the ICU?	No	8(9.8)	31(96.9)	*X*^*2*^ = 1.39 *P* = 0.441
	Yes	68(82.9)	1(3.1)	
4. Is it possible to develop the role of nursing in the ICU?	No	14(17.1)	30(93.8)	*X*^*2*^ = 2.23 *P* = 0.228
	Yes	77(93.9)	2(6.3)	
5. Do you think that ICU nurses are able to safely and correctly prescribe Medication for patients after fulfilling the requirements?	No	5(6.1)	32(100)	*X*^*2*^ = 4.83 *P* = 0.320

Also, the results of this study indicated that prescription without physician’s permission was more frequent among nurses with master’s degrees compared with those with bachelor’s degrees (X^2^ = 8.08, *p* = 0.004) (Table 5). No significant relationship was detected between drug prescription without the physician’s permission and the nurses’ working experience (Table 4).

## Discussion

The present cross-sectional study was performed to investigate the viewpoints of Iranian nurses who work in ICUs on the necessity and barriers to developing the nurse prescribing role.

Regarding the attitude of ICU nurses towards the development of the nurse prescribing role, the overall results of this section reflected the positive attitude of nurses, and nearly all of the answers showed positive effects and supported the necessity of developing this role in the ICU. Moreover, the majority of nurses considered it necessary to implement the nurse prescribing role in the ICU in response to the general question addressing the necessity of implementing this role in the ICU.

Other studies also reported positive attitudes. Clancy (2011) used a valid and reliable 31-item three-sectioned questionnaire to examine the attitudes of 45 emergency nurses regarding nurse prescribing in Ireland. Results of the study revealed that the emergency nurses maintained a positive attitude towards the development of nurse prescribing roles in the future [22]. Kroezen et al. (2014) investigated the viewpoints of licensed nurses, specialist nurses, and physicians on nursing prescribing and indicated a relatively positive attitude among nurses and physicians regarding nurse prescribing [18]. Ling et al. (2018) conducted a systematic review of the available literature and determined the necessity of development and the possibility of implementing the nurse prescribing role in China. Moreover, the results of these researches indicated the positive attitudes of the nurses regarding nurse prescribing [23]. The results of LockWood et al. (2008) on the attitude of specialist clinical nurses in Ireland indicated similar results. The majority of nurses had a positive attitude regarding the development of nurse prescribing in the future [21]. In 2005, the British Health Ministry provided a framework for advanced nursing practice in intensive care units. The necessity of this role was described and reaffirmed in intensive care units in 2006 [24, 25]. No inconsistent study was found. Though, Zarzeka et al. (2019) investigated the physicians’ attitudes regarding the expansion of professional competencies related to nursing and midwives’ prescribing and their readiness in Poland. This study indicated that physicians believed that midwives and nurses were not ready to prescribe medication, and only a small group of nurses and midwives were capable of re-prescribe physicians’ prescriptions [26]. The majority of studies demonstrated that it is vital to address the nurse prescribing subject, particularly in ICUs. In the current study, most of the participants believed that nurse prescribing has positive effects and would result in the advancement of nursing professions, which will provide comprehensive care, easy access to healthcare services, improve the quality of the healthcare, utilization of nurses’ skills, and enhanced professional independence. In line with this study, another study examined the attitudes of both physicians and nurses in this regard, both of which believed that nurse prescribing results in enhanced nursing independence [18]. LockWood et al. (2008) indicated that nurse prescribing provides easy and faster access to healthcare, improves the quality of healthcare, and results in better use of the professional skills of nurses and their increased independence [21]. Also, Zarzeka et al. (2017) revealed that nurses had a positive attitude towards the effects of nurse prescribing on patients and more than 60 % of them agreed that it could reduce the access time to health care services and improves health care access, and most of them believed that it would help patients to save time [27]. Another study in Ireland stated that nurse prescribing positively affected professional and clinical nursing development. The implementation of the nurse prescribing role attracted and retained nursing staff in the country as an essential factor, improved people’s access to the required pharmaceutical services, and provided safe and effective health care [28]. Moreover, another study shows that lack of authority may limit the provision of comprehensive care by specialized nurses [29]. Considering the various reports of the conducted studies in this regard and the reported positive effects of the nurse prescribing role implementation in the leading countries, it appears that the legitimacy of this role will be helpful for both patients and the health care system.

In the present study, most nurses believed that this role is medical profession-driven. However, in LockWood et al. (2008), more than two-thirds of participating nurses presumed the opposite. Additionally, the advantages and disadvantages of giving this role to specialized nurses were similar in the opinion of the nurses. Yet, most participants believed that it should only be assigned to nurses with any level of nursing education [21]. Contrary to this attitude, results of a qualitative study by Connor et al. (2019) in Ireland showed that the inclusion of prescription courses in the post-graduate curricula such as adult and pediatric emergency master’s degrees and intensive care master’s courses might improve the number of prescribing nurses, allowing specialized nurses the prescription license and the ability to perform an accurate assessment in a particular field [30]. Generally, no guidelines exist on the level of education required to take the advanced and specialized nursing plans. Yet, recommendations suggest that these roles may be given to nurses with at least a master’s degree [31]. Given that in most countries, specialist nurses and nurse practitioners are licensed in ICUs under advanced clinical practice [32, 33], it appears that the level of education and clinical expertise play essential roles in adopting safe and effective measures.

In our study, more than two-thirds of the participants considered themselves eligible for prescribing medication. On the other hand, another research on 644 general nurses in the Netherlands indicated that 88 % of the participating nurses believed they were not eligible for prescribing medication. This result was related to a lack of sufficient knowledge of prescribing medication [34]. Weglicki et al. (2015) conducted a qualitative study in which most participating nurses believed that their pharmaceutical knowledge was not sufficient. These nurses were worried about making wrong decisions [35]. The patients admitted to ICU face complex and unpredictable situations. These patients often receive various medications, and the safety and effectiveness of these medications should be considered [36]. Thus, the nurses’ preparedness is vital to prescription, and their eligibility is among the critical factors for the successful implementation of nursing prescribing. When deciding on licensing the nurses to prescribe medications, academic qualifications and work experience should be accurately considered [23]. The most frequently mentioned barriers regarding the attitudes towards the nurse prescribing in ICU consisted of lack of legitimacy, the opposition of physicians, lack of support by nursing managers, lack of education and knowledge, fear from the legal consequences, lack of clinical experience, not considering rewards and benefits, and lack of financial support. The responses of the participating nurses demonstrated that the lack of legitimacy is the issue upon which the majority of participating nurses agreed. Despite the mentioned barriers, most nurses stated that it is possible to implement this role for the ICU nurses. Thus, it is possible to implement nurse prescribing in the ICU through planning and overcoming the potential barriers. LockWood et al. (2008) explained that Irish nurses also indicated that lack of legitimacy is a significant barrier to overcome and reduce the fear of the legal consequences [21]. This attitude seems rational since nurse prescribing is associated with a significant increase in their legal and professional responsibilities [26]. In the current study, most participants considered the lack of physicians’ and nursing managers’ support as a critical barrier. Connor et al. (2019) demonstrated that nurse prescribing faces barriers, which are mostly related to the nursing profession. Implementing this role requires the nursing managers’ and nurses’ full support to fully reach its potential since nursing managers can determine the educational and legal requirements by recognizing the facilitators of implementation of this role. Lack of appropriate identification of these necessities acts as a barrier to implementing this role [30]. Both studies revealed that lack of financial resources is a less critical barrier to the implementation of nurse prescribing. More than half of the nurses participating in the present study were familiar with the ‘nursing prescribing’ term. Moreover, their familiarity level was related to their educational level. In other words, nurses with master’s degrees showed higher familiarity compared to nurses with bachelor’s degrees. This difference may be attributed to their familiarity with the research methods in their post-graduate studies and reviewing relevant articles to this category. No similar study was found to investigate this issue.

In the present study, more than two-thirds of the participating nurses stated that they had prescribed medications for their patients without the permission of a physician. Since this study investigated ICU nurses, the unauthorized prescription was due to the critical situations of the patients to save them. Gerard et al.’s study (2015) in the UK revealed that even in the countries where nurse prescribing is not authorized, nurses were forced to prescribe medications in emergencies in ICU to save lives [37]. Most studies in this area suggest that the majority of the nurses are attentive in the development of this role since it is formalization and publication of what they have unofficially done for several years [17–19]. Nursing care specialization requires the expansion of nursing authority and would lead to beneficial effects for patients.

### Limitations

We encountered some limitations due to the cross-sectional study design. The correlation between the studied variables remained undetermined. The other restriction of our study was related to the data collection method. Due to the self-declaration of the answers to the questionnaire, the answers may not be verified. However, the researcher attempted to overcome this limitation by explaining the objectives of this study to the participants and highlighting the anonymity of the participants.

## Conclusions

This study indicated the highly positive attitude of Iranian nurses towards the necessity of developing the nurse prescribing role. They considered it as legalization and formalization of an act that currently occurs informally by many nurses. Various barriers exist to creating such a role, the most important of which are lack of legislation, the opposition of physicians, and lack of nursing managers’ support. The nurse prescribing may be implemented (particularly in ICUs) through planning, overcoming the potential barriers, and providing the required infrastructure. Similar researches are recommended in other wards; also, we suggest investigating the viewpoints of ICU physicians on the development of the nurse prescribing role.

## Data Availability

The identified datasets analyzed during the current study are available from the corresponding author on reasonable request.
